# Multiarm studies and how to handle them in a meta‐analysis: A tutorial

**DOI:** 10.1002/cesm.12033

**Published:** 2023-12-20

**Authors:** Emma Axon, Kerry Dwan, Rachel Richardson

**Affiliations:** ^1^ Cochrane Methods Support Unit, Evidence Production and Methods Department Cochrane London United Kingdom of Great Britain and Northern Ireland; ^2^ Centre for Evidence Based Medicine, Department of Clinical Sciences Liverpool School of Tropical Medicine Liverpool United Kingdom of Great Britain and Northern Ireland

## Abstract

This tutorial focuses on multiarm studies. We will explain what multiarm studies are and how to include data from them in a meta‐analysis.

## Graphical abstract

This tutorial focuses on multi‐arm studies. We will explain what multi‐arm studies are and demonstrate how to include data from them in a meta‐analysis. In addition, we have created a micro learning module to accompany this article which includes real‐life examples of multi‐arm studies. There is also the opportunity to test out the two main approaches used to handle these study types in a meta‐analysis. Multi‐arm studies micro learning module

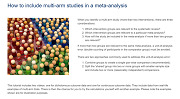

## 1 WHAT IS A MULTIARM STUDY?

A multiarm study is a study which includes more than two interventions (arms).

## 2 WHAT DO WE NEED TO CONSIDER WHEN HANDLING MULTIARM STUDIES?

When faced with a multiarm study there are three separate issues to consider:

1. Which intervention groups are relevant to the systematic review?
2. Which intervention groups are relevant to a particular meta‐analysis?
3. How will the study be included in the meta‐analysis if more than two groups are relevant?

Some arms of a multiarm study might not be relevant to the review. For example, a systematic review comparing topical interventions versus placebo for eczema might identify a trial which has three arms: a systemic treatment, a topical treatment, and placebo. The systemic treatment arm is not relevant to the review so the review authors can ignore the data from this arm and treat the study as a standard two‐arm trial.

However, if this systematic review included both systemic and topical treatments then all three arms would be of interest. Review authors will then need to consider how they have planned to structure their syntheses to answer the review questions. If the review authors plan to analyse topical treatments separately from systemic treatments, then they would include the two intervention arms in separate meta‐analyses, compared against placebo.

However, there may be occasions where both intervention arms are relevant to include in a single meta‐analysis. For example, there might be a review looking at dietary interventions to prevent obesity. A trial has three arms—dietary intervention via face‐to‐face delivery, dietary intervention via an online interactive website, or no intervention. If the authors include both intervention arms against the “no intervention” group in the meta‐analysis, they would be including the “no intervention” group twice (usually referred to as “double counting” of participants). This creates a “unit of analysis” error where the meta‐analysis fails to address the correlation between the estimated intervention effects from multiple comparisons. To overcome this issue, it may be considered appropriate to pool the two dietary interventions into one group.

## 3 WHAT APPROACH SHOULD REVIEW AUTHORS TAKE WHEN INCLUDING MULTIARM STUDIES IN A META‐ANALYSIS?

There are two main approaches to handle this scenario. First, we shall look at dichotomous outcome data.

### 3.1 Combine groups to create a single pairwise comparison (dichotomous outcome data)

The recommended way to deal with this problem is to combine the two groups, which overcomes the unit‐of‐analysis error, creating a single pair‐wise comparison. Table 1 shows a hypothetical example of dichotomous outcome data (number of participants who lost weight at 6 months follow up), extracted from a trial comparing two dietary interventions to a control group.

**Table 1:** Dichotomous data from a hypothetical three‐arm trial.

	Dietary intervention (face‐to‐face)	Dietary intervention (online)	No intervention
Number of people who lost weight	21	15	10
Total number of people	49	47	52

**Figure 1:**
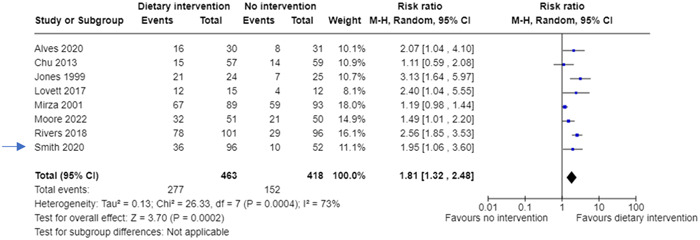
Forest plot showing a meta‐analysis including data from a hypothetical three‐arm trial (Smith, 2020) where the two interventions have been combined.

Review authors can combine the data from the two intervention groups to create a new 2 × 2 table (Table 2). This way all the data is included and there is no risk of “double counting” the control group. The forest plot in Figure 1 shows how these hypothetical data (as “Smith 2020”) would be presented in a forest plot. But the potential disadvantage of this method is that the readers do not see the data split by type of dietary intervention, and this may be of interest.

**Table 2:** Dichotomous data from a hypothetical three‐arm trial: data from two intervention arms combined.

	Dietary interventions (face‐to‐face/online)	No intervention
Number of people who lost weight	36	10
Total number of people	96	52

### 3.2 Split the comparison group into two or more groups (dichotomous outcome data)

**Figure 2:**
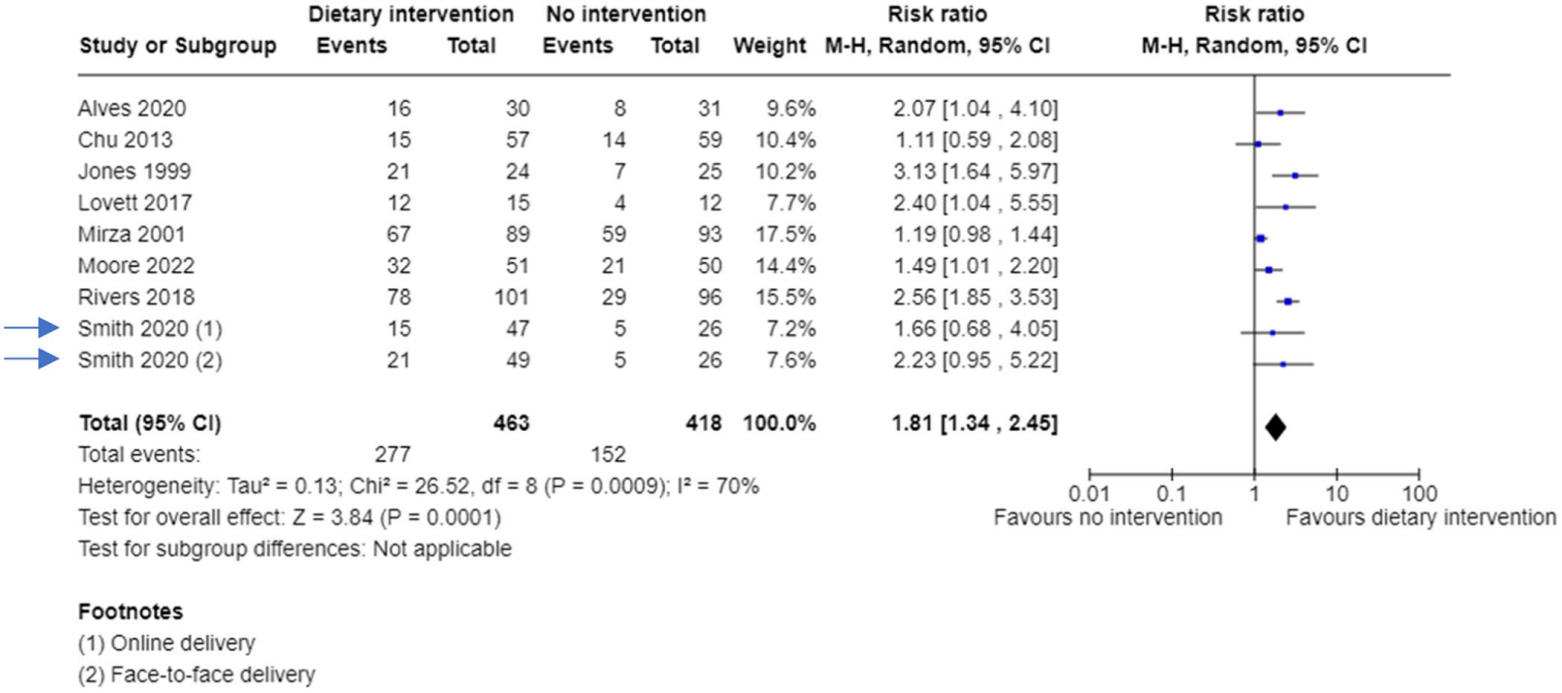
A forest plot showing a meta‐analysis including data from a hypothetical multiarm study (Smith, 2020), where the events and participants in control group has been halved.

The other option is to split the “shared” group into two or more groups with a smaller sample size and include two or more (reasonably independent) comparisons. This method isn't usually recommended over option 1 because the comparisons remain correlated, so this only partially accounts for the unit‐of‐analysis error. But it may be favored if the review authors want to investigate intervention‐related sources of heterogeneity. Further details are given in Chapter 23 of the Cochrane Handbook [1]. Table 3 shows two new 2 × 2 tables created from the same hypothetical data shown in the first example, where the number of events (*n* = 10) and the number of people in the control group (*n* = 52) have been halved, to avoid a unit‐of‐analysis error (“double counting”). Figure 2 shows how these data would look in a forest plot, where the two comparisons can be shown in the same meta‐analysis. They could also be included a subgroup analysis.

**Table 3:** 2 × 2 tables created from hypothetical dichotomous data, showing the “no intervention” data split into two groups.

Analysis 1	Dietary intervention (face to face)	No intervention
Number of people who lost weight	21	5
Total number of people	49	26

**Analysis 2**	**Dietary intervention (online)**	**No intervention**
Number of people who lost weight	15	5
Total number of people	47	26

The combining and the splitting approaches generate the same value for the relative risk (1.81) but a slightly different 95% confidence interval.

## 4 HOW IS CONTINUOUS OUTCOME DATA HANDLED?

**Figure 3:**
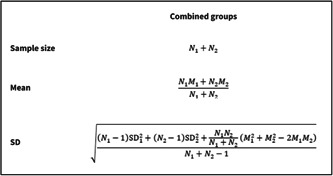
Formulae used to calculate the sample size, mean, and standard deviation (SD) when combining continuous data from two groups (table 6.5.a, Cochrane Handbook [2).

**Figure 4:**
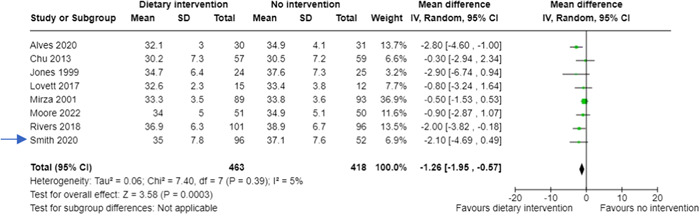
Forest plot showing a meta‐analysis including continuous outcome data from a hypothetical three‐arm trial (Smith, 2020) where the two interventions have been combined.

However, what approach do review authors take when the outcome data is continuous (e.g., body mass index at 6 months follow‐up)? To combine the data from the two dietary interventions in a hypothetical example (Table 4), review authors need to use the formulae (Figure 3) in Chapter 6.5 of the Cochrane Handbook [2]. This results in the means, standard deviations (SDs), and sample sizes from the two intervention groups being combined (Table 5). These data can then be entered into a forest plot (Figure 4).

**Table 4:** Continuous outcome data from a three‐arm trial.

	Dietary intervention (face‐to‐face)	Dietary intervention (online)	No intervention
BMI (SD)	35.2 (7.5)	34.8 (8.2)	37.1 (7.6)
Total number of people	49	47	52

Abbreviation: BMI, body mass index.

**Table 5:** Continuous outcome data from a hyopthetical three‐arm trial, where data from the intervention groups have been combined.

	Dietary interventions (face‐to‐face/online)	No intervention
BMI (SD)	35.0 (7.8)	37.1 (7.6)
Total number of people	96	52

Abbreviation: BMI, body mass index.

**Figure 5:**
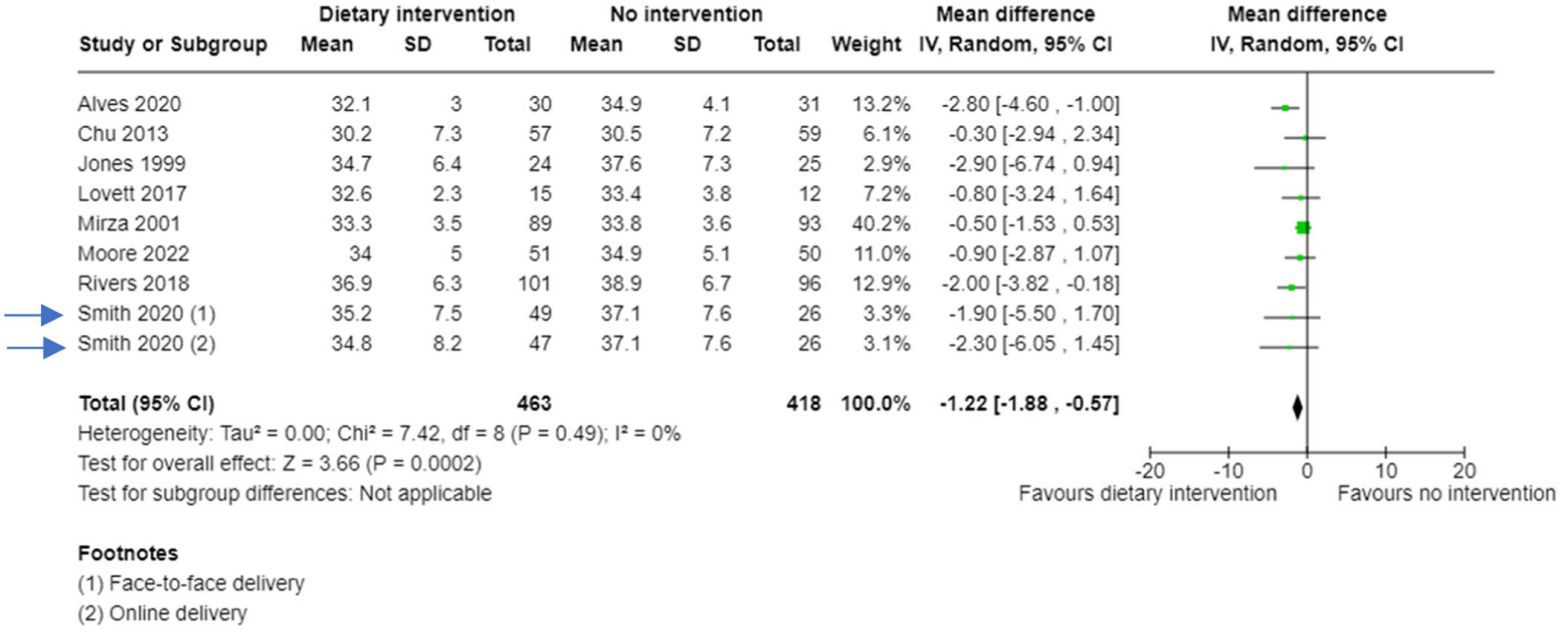
Forest plot showing a meta‐analysis including continuous outcome data from a hypothetical three‐arm trial (Smith, 2020) where the control group has been halved.

If the authors want to use the approach of splitting the comparison group into two or more groups for continuous data, then they simply use the same mean (SD) for the “no intervention” group but halve the number of participants in this group (*n* = 26 in each “control” group). Figure 5 shows the inclusion of such data in a forest plot; a subgroup analysis could also be performed.

As with the dichotomous outcome data, the combining and the splitting approaches make very little difference to the overall effect size and confidence interval.

## 5 ARE THERE ANY OTHER OPTIONS AVAILABLE?

Another option is to include two or more correlated comparisons and account for the correlation. In each relevant pair‐wise analysis, a weighted average can be calculated along with a variance (weight) for the study. This considers the correlation between comparisons [3]. However, this method typically produces a similar result to the method of combining the groups to create a single pair‐wise comparison, so it's not regularly used in favor to the other methods described above (which are simpler to conduct).

A final option is to undertake a *network meta‐analysis* [4]. This method allows for the correlation between groups, so it is possible to include all relevant arms of a study in the network without any double‐counting issues. However, a network meta‐analysis is a more complicated method and needs careful planning and input from an experienced statistician.

## 6 FURTHER READING AND ONLINE CONTENT

More information on multiarm studies can be found in Chapter 23 of The Cochrane Handbook for Systematic Reviews of Interventions [1].

**Figure 6:**
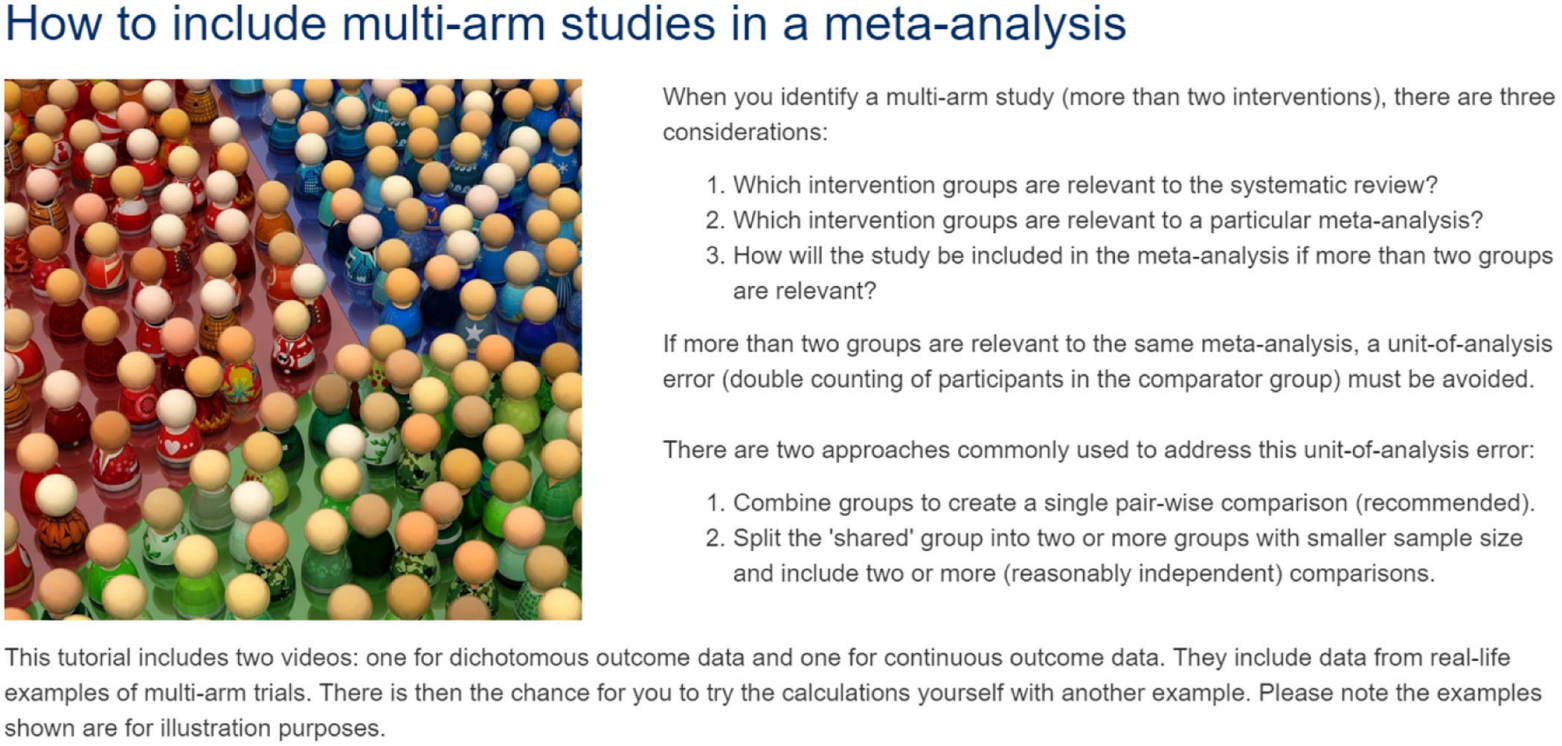
Screenshot of the micro‐learning module.

Cochrane Training has produced a micro‐learning module on how to include data from multiarm studies in a meta‐analysis to accompany this article (Figure 6) [5].

## AUTHOR CONTRIBUTIONS

**Emma Axon**: Conceptualization; methodology; project administration; writing—original draft; writing—review and editing. **Kerry Dwan**: Conceptualization; supervision; writing—review and editing. **Rachel Richardson**: Conceptualization; supervision; writing—review and editing.

## CONFLICT OF INTEREST STATEMENT

Emma Axon and Rachel Richardson are employed by Cochrane. Kerry Dwan is a former employee of Cochrane.

## Data Availability

Data sharing not applicable to this article as no data sets were generated or analyzed during the current study. The data used in this article is ‘hypothetical’.
